# Action potentials induce biomagnetic fields in carnivorous Venus flytrap plants

**DOI:** 10.1038/s41598-021-81114-w

**Published:** 2021-01-14

**Authors:** Anne Fabricant, Geoffrey Z. Iwata, Sönke Scherzer, Lykourgos Bougas, Katharina Rolfs, Anna Jodko-Władzińska, Jens Voigt, Rainer Hedrich, Dmitry Budker

**Affiliations:** 1 Helmholtz Institute Mainz, GSI Helmholtzzentrum für Schwerionenforschung, Darmstadt, Germany; 2 Johannes Gutenberg University of Mainz, Mainz, Germany; 3 Department of Molecular Plant Physiology and Biophysics, University of Würzburg, Würzburg, Germany; 4 Physikalisch-Technische Bundesanstalt, Berlin, Germany; 5 Faculty of Mechatronics, Warsaw University of Technology, Warsaw, Poland; 6 Department of Physics, University of California, Berkeley, CA USA

**Keywords:** Biophysics, Plant sciences, Physics

## Abstract

Upon stimulation, plants elicit electrical signals that can travel within a cellular network analogous to the animal nervous system. It is well-known that in the human brain, voltage changes in certain regions result from concerted electrical activity which, in the form of action potentials (APs), travels within nerve-cell arrays. Electro- and magnetophysiological techniques like electroencephalography, magnetoencephalography, and magnetic resonance imaging are used to record this activity and to diagnose disorders. Here we demonstrate that APs in a multicellular plant system produce measurable magnetic fields. Using atomic optically pumped magnetometers, biomagnetism associated with electrical activity in the carnivorous Venus flytrap, *Dionaea muscipula*, was recorded. Action potentials were induced by heat stimulation and detected both electrically and magnetically. Furthermore, the thermal properties of ion channels underlying the AP were studied. Beyond proof of principle, our findings pave the way to understanding the molecular basis of biomagnetism in living plants. In the future, magnetometry may be used to study long-distance electrical signaling in a variety of plant species, and to develop noninvasive diagnostics of plant stress and disease.

## Introduction

In the plant kingdom, electrical signaling pathways are involved in reception and transduction of external stimuli such as light^1^, temperature^2^, touch^3,4^, wounding^5^, and chemicals^6^. Although human and animal magnetophysiology are well-developed areas of research^7–15^, very little analogous work has been conducted in the plant kingdom^1,5,16,17^. Our research aims to help establish magnetic sensing as a viable complement to traditional plant-electrophysiological techniques^18^.

**Figure 1:**
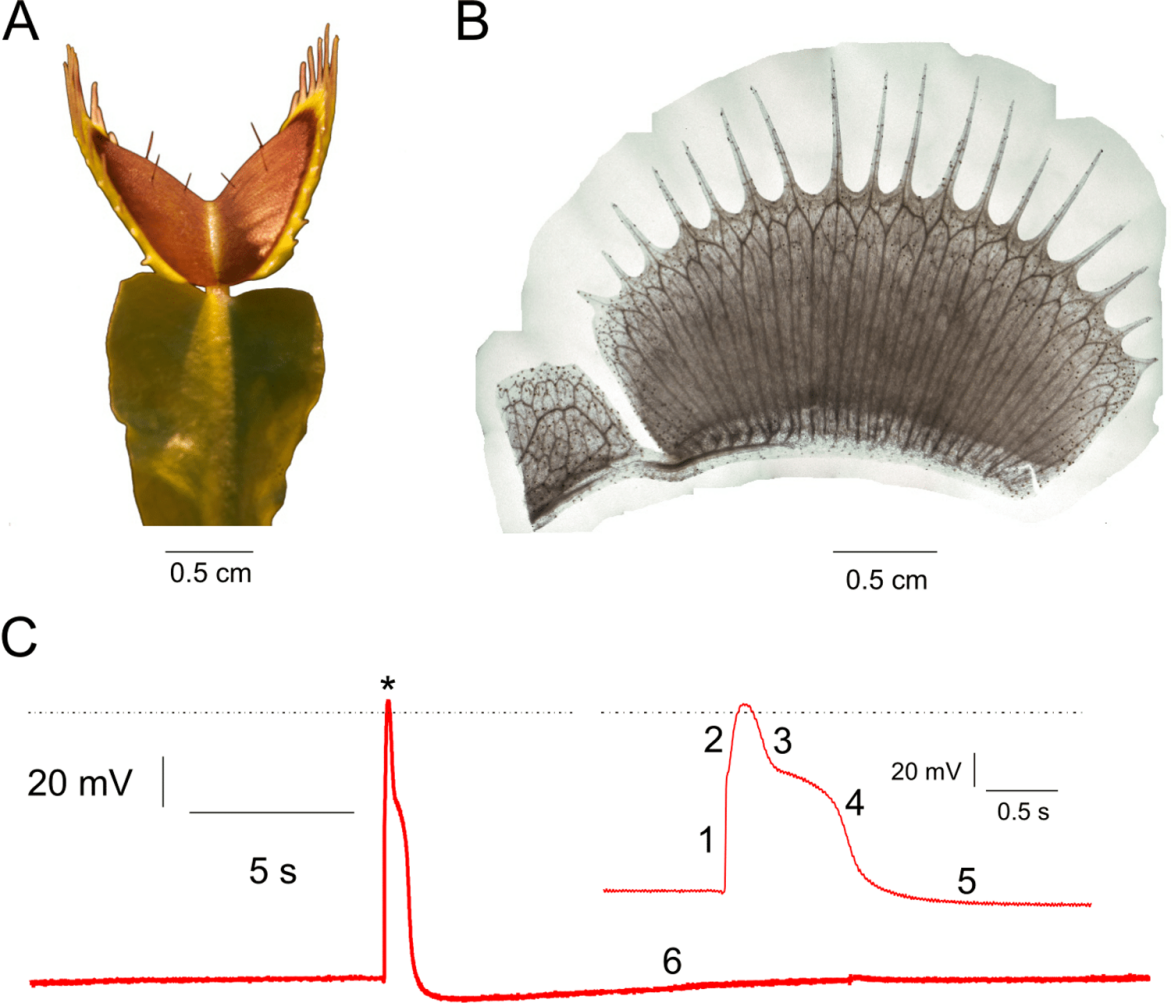
Venus flytrap geometry and action potentials. (**A**) *Dionaea muscipula* leaf forms into a bivalved snap trap connected to the leaf stalk, or petiole. (**B**) Side view of a destained trap lobe showing vasculature structure. In contrast to the petiole, the trap contains parallel veins of interconnected cells. These veins consist of both dead low-conductivity water pipes (xylem) and living conductive phloem. Here the trap was destained using a modified protocol according to^26^, as described in Methods. (**C**) Intracellular AP lasting 2 s is subdivided into six phases (numbers), as explained in the text. The depolarization peak is indicated by an asterisk; the dotted line represents 0 mV. Inset: zoom-in on the AP, resolving the first five phases of the AP.

The bilobed trap of the *Dionaea muscipula* plant^4,19^ (Fig. 1A,B), formed by the modified upper part of the leaf, snaps closed within a fraction of a second when touched. Three trigger hairs that serve as mechanosensors are equally spaced on each lobe. When a prey insect touches a trigger hair, an AP (Fig. 1C) is generated and travels along both trap lobes^20^. If a second touch-induced AP is fired within 30 s, the viscoelastic energy stored in the open trap is released and the capture organ closes^21,22^, imprisoning the animal food stock for digestion of a nutrient-rich meal. The leaf stalk, or petiole, is not excitable and is electrically insulated from the trap^23^. Because of this, the trap can be isolated functionally intact from the plant by a cut through the petiole. On the isolated trap, stimuli trigger APs and closure just as on the intact plant. For the comfort of electrophysiological studies, one of the trap lobes can be fixed to a support while the other is removed, without affecting the features of AP firing. It has been shown that this simplified experimental flytrap system is well-suited to study the AP under highly reproducible conditions^24^. Other than by touch (mechanical energy), APs in traps can be induced by salt loads (osmotic energy)^25^ and temperature changes (thermal energy).

Since mechanical activation of APs can cause unwanted noise in electric and magnetic recordings, we use thermal stimulation in our experiments. The interdisciplinary work presented here encompasses two complementary sets of experiments: the temperature dependence of flytrap electrical activity was studied in a plant-physiology laboratory, while magnetometer measurements of heat-stimulated traps were conducted in a magnetically shielded room.

## Heat-induced action potentials

**Figure 2:**
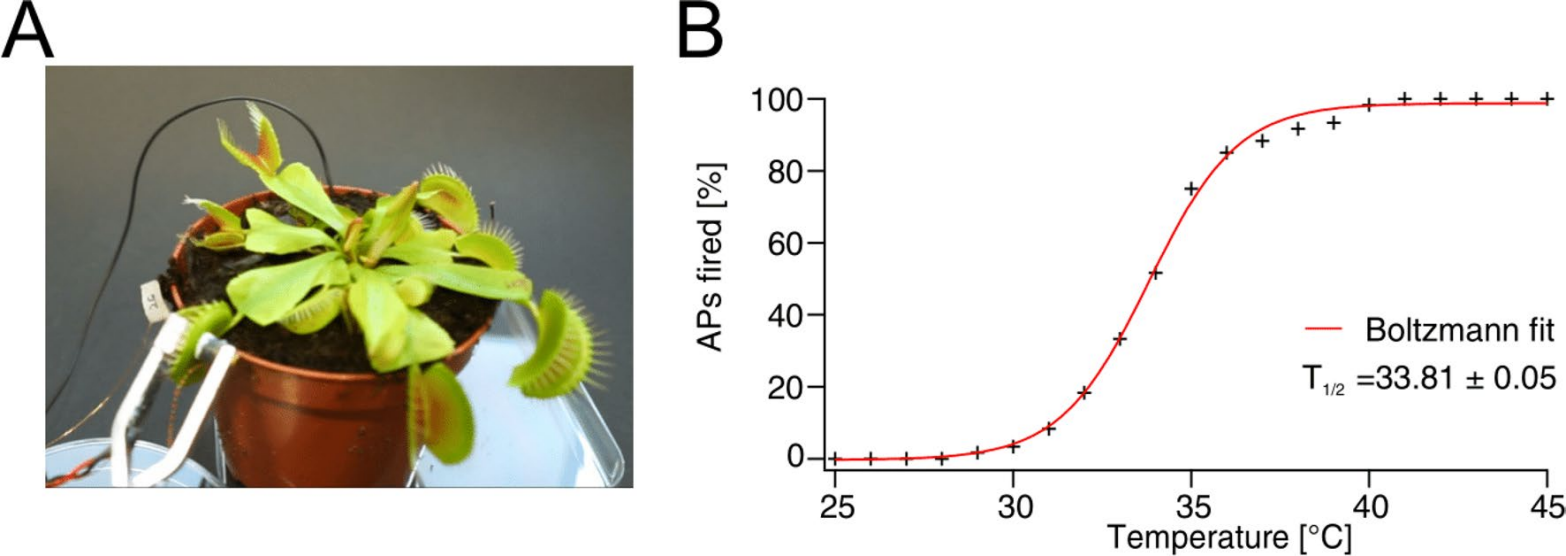
Electrical measurements of heat-induced action potentials. (**A**) *Dionaea* plant with clamp mounted on one lobe of a trap, equipped with a Peltier device and surface-voltage electrode. A ground electrode is placed in the soil surrounding the plant root. (**B**) Temperature dependence of AP-firing probability fitted by a Boltzmann equation (red curve), characterized by 50% firing probability at temperature *T*_1/2_.

When we heated up the support to which excised open traps were fixed, APs were elicited and the traps closed (Supplementary Information, Fig. S1 and Movie S1). To study the temperature dependence of heat-induced AP initiation (Fig. 2A), from a resting temperature of 20 °C, the trap temperature was increased monotonically to 45 °C at a rate of 4 °C/s (Fig. S2). Below 30 °C, no APs were observed; above 30 °C, the probability of AP firing increased and was maximal (100%) above 40 °C. In 60 independent experiments using 10 different traps from 10 different plants, we recorded the temperature at which an AP was first induced. When these data were plotted as temperature-dependent AP-firing probability (Fig. 2B), the curve could be well-fitted by a single Boltzmann equation characterized by a 50% AP-firing probability at 33.8 °C. This behavior indicates that heat activation of the AP is based on a two-state process. The ion channels that carry the classical animal-type AP also occupy two major states: closed and open. In contrast to the animal sodium-based AP, the plant AP depolarization is operated by a calcium-activated anion channel^4^. Thus, we conclude that the temperature “switch” of the Dionaea AP is based on a calcium-dependent process. Following Ca^2+^ binding, the anion-channel gates open. Our experiments indicate that at temperatures of *T* ≲ 34 °C the cellular Ca^2+^ level remains below threshold, but at *T* ≳ 34 °C there is enough chemical energy to open a critical number of anion channels, driving the fast depolarization phase of the AP.

The *Dionaea* AP can be subdivided into 6 well-defined phases (Fig. 1C): (1) fast depolarization, (2) slow depolarization, (3) fast repolarization, (4) slow repolarization, (5) transient hyperpolarization, and (6) slow recovery of the membrane potential to the pre-AP state. When comparing APs recorded at different temperatures, we found that temperature affects the signal amplitude and duration. Increasing the thermal energy input changed not only the probability for an AP to be fired, but also led to an increased AP amplitude and decreased half-depolarization time (Supplementary Information). These facts indicate that heat-sensitive ion channels trigger and shape the AP: at higher temperatures, thermal energy input causes more closed Ca^2+^-activated anion channels to open and depolarize the membrane potential. Compared to depolarization, fast repolarization (mediated by K^+^ channels) and transient hyperpolarization (caused by depolarization activation of outward-directed protein pumps) were much less affected by temperature. The recovery time to reach the resting membrane potential was essentially insensitive to temperature changes.

Besides lowering the AP firing threshold and changing certain features of the AP, prolonged heat stimulation can induce trap lobes to enter an autonomous AP firing mode (Fig. S1). When increasing the bottom surface temperature of the recording-chamber base from 20 to 46 °C, AP spiking activity sets in after a couple of seconds, reaching a steady AP firing frequency of 3.8 per minute at a stable 46 °C surface temperature. Induction of autonomous APs has also been obtained using flytraps treated with NaCl salt (osmotic energy)^27^.

## Biomagnetism

**Figure 3:**
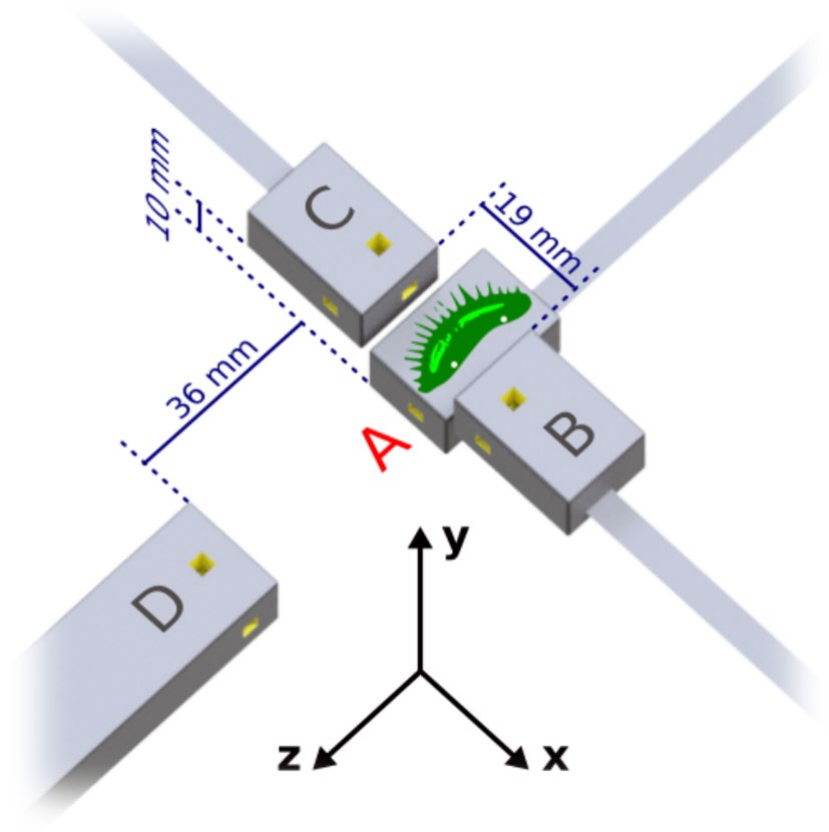
Schematic of the experimental setup in the magnetically shielded room. The plant sample, an isolated lobe of the flytrap, is placed on top of primary sensor A, in the *x*–*z* plane with trigger hairs exposed. For reference, the dimensions of the housing (gray boxes) for the primary and secondary sensors are 24.4 × 16.6 × 12.4 mm^3^. Yellow cut-outs indicate the position of the 3 × 3 × 3 mm^3^ atomic sensing volume. A 3D-printed ABS plastic structure (not shown) holds the magnetometers in position on a wooden table. White dots on the plant sample, approximately 1 cm apart, indicate the placement of surface electrodes for AP monitoring. In the coordinate system shown, all magnetometers are sensitive along the *y*-axis, normal to the surface of the plant sample; furthermore, A and D are sensitive along the *z*-axis, and B and C are sensitive along the *x*-axis. Sensors B and C are positioned symmetrically around sensor A. Sensor D serves as a background sensor and is therefore located farther away from the sample.

Having established heat stimulation as a reliable noninvasive technique for inducing flytrap APs, we searched for the magnetic field associated with this electrical excitability. Magnetometry experiments were carried out at Physikalisch-Technische Bundesanstalt (PTB) Berlin in the Berlin Magnetically Shielded Room 2 (BMSR-2) facility^28^, using four QuSpin Zero-Field Magnetometers (QZFM). These commercial optically pumped magnetometers (OPMs) employ a glass cell containing alkali vapor to sense changes in the local magnetic-field environment^29,30^. A magnetically shielded environment is required for operation of these magnetometers, and use of a walk-in shielded room allowed for the constant presence of an experimenter to prepare plant samples and carry out measurements. As shown in Fig. 3, an isolated trap lobe was attached to the housing of the primary sensor (denoted A), such that the distance between the plant sample and the center of the atomic sensing volume was approximately 7 mm. Two secondary sensors (B and C) were placed nearby the primary sensor to measure signal fall-off, and an additional background sensor (D) was used to monitor the magnetic environment in the shielded room. Each magnetometer is sensitive to signals along two orthogonal axes. Resistive heaters in the magnetometer housing, which are used to increase the atomic density and improve sensitivity, also served to induce autonomous AP firing via surface heat transfer at 41 °C. To monitor heat-induced APs, we used two silver-tipped copper surface electrodes, inserted in either end of the plant sample^31^. Prior to the measurements, we performed tests to ensure that no spurious magnetic fields were generated by the electrode system (Supplementary Information, Figs. S3 and S4). The magnetic and electric data, together with other auxiliary trigger signals, were sampled simultaneously using the same data-acquisition system located outside the magnetically shielded room.

**Figure 4:**
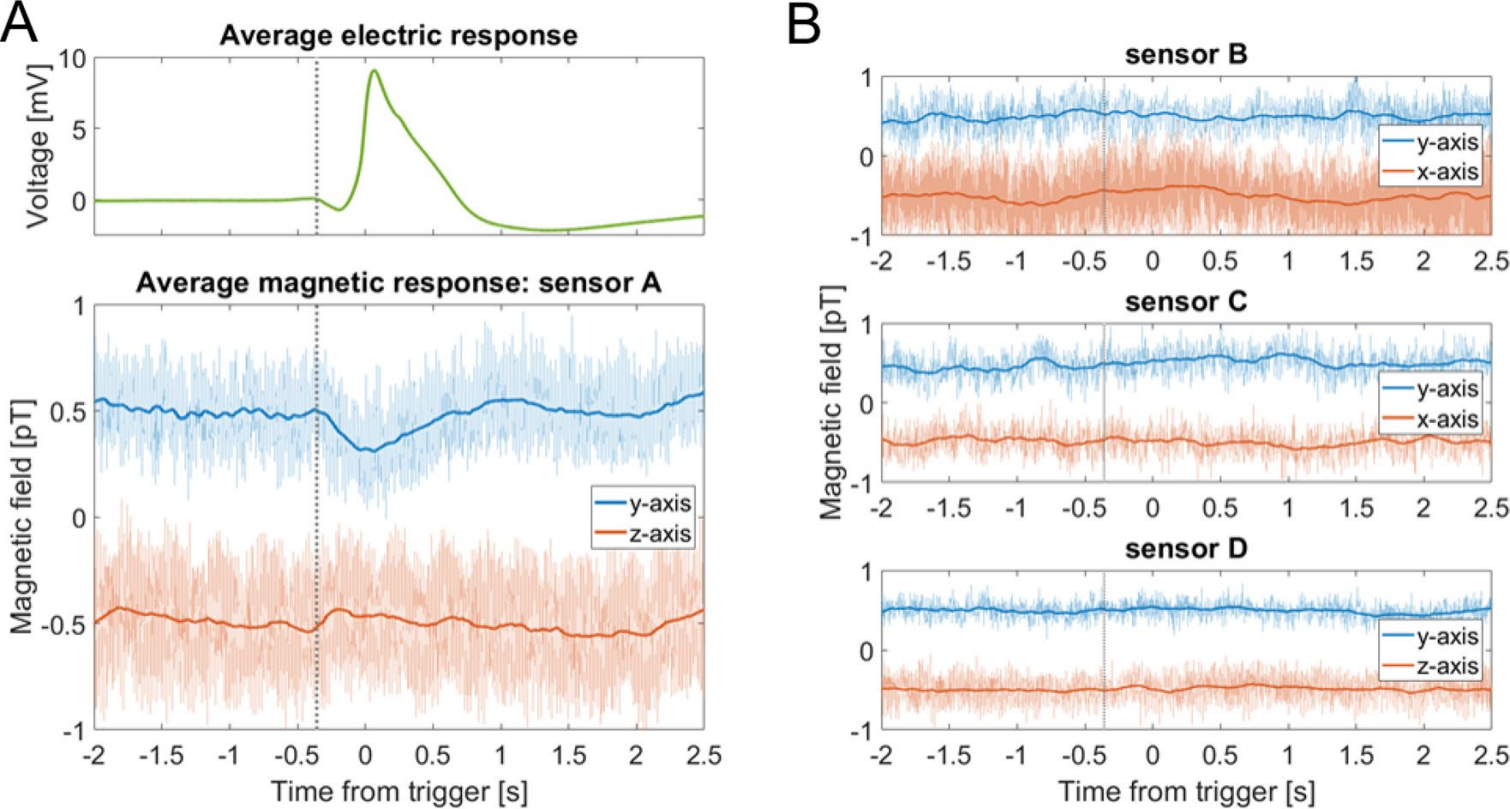
Average action potential and corresponding magnetic signals. (**A**) Result of triggering on nine consecutive APs from a trap lobe heated to 41 °C, then averaging the electric and magnetic data from a 4.5 s window around each trigger point. The average magnetic traces (bottom graph, opaque traces) were frequency-filtered (50 Hz low-pass), then smoothed with a 0.2 s running average. A magnetic signal is visible in both sensitive axes of the primary sensor A. For comparison, the raw unfiltered data are plotted behind the processed data. For visual clarity, DC offsets have been added to the data, and vertical gray dotted lines indicate the approximate start time of the electric signal. (**B**) Average magnetic response from the other three sensors, obtained using the same procedure as in (**A**). The data from the secondary sensors, B and C, do not show a signal. The data from the background sensor D can be used to remove noise common to all sensors (see Fig. 5).

**Figure 5:**
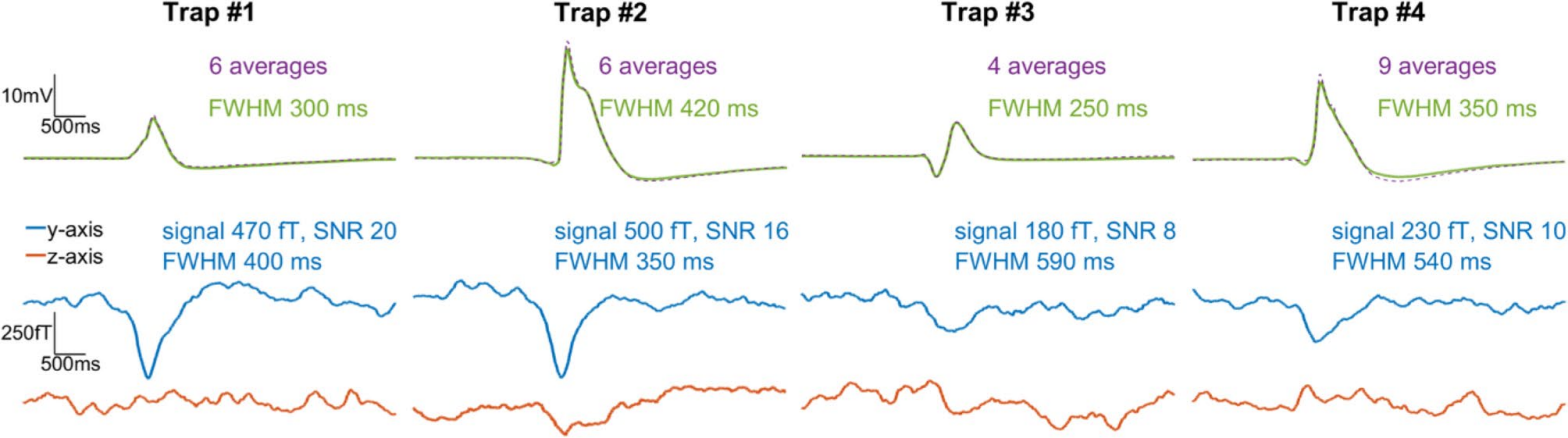
Comparison of average electric and gradiometric signals from four different experiments. In each case we triggered on heat-induced APs in the electric trace and performed the same data analysis as for Fig. 4. The electric response recorded by the surface electrodes (top row; average signal plotted as solid green, single AP plotted as dashed purple) varies in amplitude because a different plant sample was used in each experiment. To produce the gradiometric plots (bottom graphs) we subtracted the magnetic data of background sensor D from that of primary sensor A. The number of averages in each experiment is indicated, along with the amplitude and signal-to-noise ratio (SNR) of the *y*-axis gradiometric signal. The rightmost panel (Trap #4) shows the same data set as in Fig. 4.

To better distinguish possible magnetic signals from background noise, we triggered on the electric signals and averaged the magnetic data in a time window around those trigger points. Examples of averaged magnetic data are shown in Fig. 4. A clear magnetic signal with a time scale corresponding to that of the averaged electric signal is visible in the primary-sensor data. For comparison, data from several different experiments were plotted (Fig. 5). To minimize common background noise, we subtracted the magnetic data of sensor D to create a gradiometer with a 48-mm baseline. Signals of up to 0.5 pT are visible in the *y*-axis gradiometric data, normal to the sample surface. The signal magnitude obtained is comparable to what one observes in surface measurements of nerve impulses in animals^14^.

To quantify the significance of the measured signals, signal-to-noise ratios (SNRs) were calculated from the average *y*-axis gradiometric time traces as follows. The noise level is defined as the standard deviation of the gradiometric response in a 1.5 s time window (from time *t* =  − 2 s to *t* =  − 0.5 s in Fig. 4A) prior to signal onset. The signal size is defined as the amplitude of the extreme (minimum) field value, with respect to the mean value in the noise window. For the four experiments shown in Fig. 5, the SNRs range from 8 to 20. The corresponding p-values are *p* < 9 × 10^−16^, indicating that the probability of such signals arising from random noise is negligible. At the sub-Hz signal frequency, the sensitivity of the gradiometer is approximately 100 fT/√Hz (Fig. S6). For both the electric and magnetic signals, the full width at half extremum (maximum or minimum, FWHM) were also calculated, where the extremum is defined with respect to the mean value in the noise window.

The temporal superposition of the electric and magnetic signals in Fig. 5 suggests that we have detected the magnetic activity associated with the flytrap AP. Unlike in measurements of animal nerve axons and the large internodal cells of *Chara corallina* alga, where the magnetic field is proportional to the time derivative of the intracellular voltage^13,16,18^, the magnetic signal from the complex multicellular flytrap lobe has a shape similar to that of the electric signal. We see features in the magnetic signal which appear to correspond to the depolarization and repolarization phases of the AP. In electric recordings using surface electrodes, the exact shape and duration of signals are dependent on the placement of electrodes on the measured sample. By contrast, magnetometry records a “true” physical signal from the organism. In this sense, it is comparable to intracellular electrode techniques. Whereas intracellular electrodes are sensitive to electrical activity of single cells, magnetometers can record both local and systemic activity at the multicellular level. In this way, magnetometry might be considered a complementary tool to the emerging technique of gene-based imaging of plant electrical signaling^23,32^.

The physical origin of the measured biomagnetic fields is related to an outstanding question in plant electrophysiology: how electrical signals propagate over long distances through the plant. Essentially this is a scaling problem: while electrical signaling is well-understood in some unicellular plant systems^16^, much less is known about the propagation mechanisms of such signals between cells and along cellular pathways. For the Venus flytrap system, it is known from electrode measurements that APs propagate through the trap at speeds of around 10 m/s^19^. A proposed pathway of long-distance signal propagation between plant cells in the trap is the electrically conductive phloem in the vasculature (Fig. 1B). Given that the typical resistance between two points on a trap is *R* ≈ 1 MΩ^33^, we can perform a basic calculation to confirm that the magnitude of the magnetic fields we measure is reasonable. We estimate the expected magnetic-field magnitude at the center of the sensing volume to be

1 $$\begin{array}{*{20}{l}} {B \approx \frac{{{\mu_0}I}}{{2{\uppi }r}}\,,} \end{array}$$

where *I* = *V*/*R* ≈ 10 nA is the current passing through the trap between the electrodes, and *r* ≈ 7 mm is the perpendicular distance from the trap surface. Using these values, we find *B* ≈ 0.3 pT, a magnitude which corresponds well with the *y*-axis experimental results of sensor A. Although the precise distribution and directionality of current flow in the trap is unknown, we can use the geometry of the trap (Fig. 1A,B) and magnetometry setup (Fig. 3) to further interpret our results. If the *x*-oriented parallel-cable structure of the vasculature is the primary conduction pathway, magnetic field along the *y*-direction is expected at the primary sensor A, but not at the secondary sensors B and C. The symmetry of the trap about the *x*-direction could explain the relative lack of *z*-axis magnetic signal in our measurements. Thus, our magnetometry results agree with a hypothesis that the vasculature serves as a network for long-distance electromagnetic signaling within the trap.

## Discussion

Previously reported detection of plant biomagnetism, which established the existence of measurable magnetic activity in the plant kingdom, was carried out using superconducting-quantum-interference-device (SQUID) magnetometers^1,5,16^. Atomic magnetometers are arguably more attractive for biological applications, since, unlike SQUIDs^34,35^, they are non-cryogenic and can be miniaturized to optimize spatial resolution of measured biological features^14,15,36^. In the future, the SNR of magnetic measurements in plants will benefit from optimizing the low-frequency stability and sensitivity of atomic magnetometers. Just as noninvasive magnetic techniques have become essential tools for medical diagnostics of the human brain and body, this noninvasive technique could also be useful in the future for crop-plant diagnostics—by measuring the electromagnetic response of plants facing such challenges as sudden temperature change, herbivore attack, and chemical exposure.

## Methods

### Biology

To obtain strong electric and magnetic signals, the health of the plants is paramount. We purchase adult Venus flytraps from a carnivorous-plant greenhouse (Gartenbau Weilbrenner, Freinsheim, Germany). Normally the plant samples are housed in a growth chamber manufactured by Poly Klima. To keep the flytraps alive during the PTB measurement run, we used homemade plastic greenhouses equipped with plant-cultivation lighting and temperature and humidity monitoring. The plants were kept on an automated 12/12-h light/dark cycle at approximately 25 °C and 75% relative humidity, treated only with distilled water.

To produce the destained image of the flytrap lobe shown in Fig. 1B, the trap chlorophyll was removed using methanol at 37 °C for 4 weeks, followed by final clearing in 4% sodium dodecyl sulfate (SDS) for 4 weeks.

For recording of flytrap APs in our heat-stimulation investigations, we used surface electrodes measuring the extracellular potential of a trap. The measuring electrode (blank silver wire; 0.25 mm, WPI, Sarasota, USA) was inserted into the trap, with the electrical connection enhanced by application of a droplet of contact gel (Laboklinika), while the reference electrode was inserted into wet soil or the petiole midrib. Electrical signals were amplified 100-fold and recorded with Patchmaster software (HEKA). Temperature dependence of AP induction was studied by application of a homemade Peltier device powered by a PTC-10 temperature-control system (npi electronic, NJ 08,510, United States). Constant heat was applied using an IKA RET basic hot plate (IKA-Werke GmbH & Co. KG, Staufen, Germany) heated to 46 °C.

### Magnetometry

Several types of magnetometry experiments were conducted at PTB: controls, OPM measurements using four QuSpin sensors (three Gen-2: denoted A, B, C; one Gen-1.5: denoted D), and measurements using the multi-channel SQUID array of BMSR-2. See Supplementary Information for further details of the SQUID measurements.

For the OPM measurements of isolated trap lobes, each sample was cleaved from the plant with a razor blade and placed on the primary sensor A for immediate measurement. The sample was either secured to the sensor housing with double-sided adhesive tape (acrylate, thickness 0.5 mm) or placed on a plastic slide (PET, thickness 0.22 mm) on the housing. Electrode, magnetometer, and electric reference signals were recorded at a 500-Hz acquisition rate on a 9-channel analog data-acquisition system with PC control. The raw difference signal from the two surface electrodes was first sent through a voltage preamplifier (Stanford Research Systems, Model SR560), AC-coupled with a gain of 100. It is essential to use a voltage, rather than current, preamplifier to avoid currents in the electrical leads whose magnetic fields may be detected by the magnetometers. Since leakage of electrical signals into magnetic channels is a serious concern, we address the topic in detail in Supplementary Information.

## Supplementary Information

Supplementary Video.

Supplementary Information.

## Data Availability

The datasets generated and analyzed during the current study are available from the corresponding author on reasonable request.

## References

[1] Baudenbacher F, Fong LE, Thiel G, Wacke M, Jazbinšek V, Holzer JR, Stampfl A, Trontelj Z (2005). Intracellular axial current in *Chara corallina* reflects the altered kinetics of ions in cytoplasm under the influence of light. Biophys. J..

[2] Sharma P, Sharma N, Deswal R (2005). The molecular biology of the low-temperature response in plants. BioEssays.

[3] Evans M (2003). Touch sensitivity in plants: be aware or beware. Trends Plant Sci..

[4] Hedrich R, Neher E (2018). Venus flytrap: how an excitable, carnivorous plant works. Trends Plant Sci..

[5] Jazbinšek V, Thiel G, Müller W, Wübbeler G, Trontelj Z (2000). Magnetic detection of injury-induced ionic currents in bean plants. Eur. Biophys. J..

[6] Volkov AG (2000). Green plants: electrochemical interfaces. J. Electroanal. Chem..

[7] Baillet S (2017). Magnetoencephalography for brain electrophysiology and imaging. Nat. Neurosci..

[8] Slichter CP (1990). Principles of Magnetic Resonance.

[9] Glover GH (2011). Overview of functional magnetic resonance imaging. Neurosurg. Clin. N. Am..

[10] Schomer DL, Lopes da Silva FH (2017). Niedermeyer’s Electroencephalography: Basic Principles, Clinical Applications, and Related Fields.

[11] Cohen D (2004). DC magnetic fields from the human body generally: a historical overview. Neurol. Clin. Neurophysiol..

[12] Williamson SJ, Romani GL, Kaufman L, Modena I (1983). Biomagnetism: An Interdisciplinary Approach.

[13] Barry JF, Turner MJ, Schloss JM, Glenn DR, Song Y, Lukin MD, Park H, Walsworth RL (2016). Optical magnetic detection of single-neuron action potentials using quantum defects in diamond. PNAS.

[14] Jensen K, Budvytyte R, Thomas RA, Wang T, Fuchs AM, Balabas MV, Vasilakis G, Mosgaard LD, Stærkind HC, Müller JH, Heimburg T, Olesen SP, Polzik ES (2016). Non-invasive detection of animal nerve impulses with an atomic magnetometer operating near quantum limited sensitivity. Sci. Rep..

[15] Jensen K, Skarsfeldt MA, Stærkind H, Arnbak J, Balabas MV, Olesen SP, Bentzen BH, Polzik ES (2018). Magnetocardiography on an isolated animal heart with a room-temperature optically pumped magnetometer. Sci. Rep..

[16] Trontelj Z, Zorec R, Jazbinšek V, Erné SN (1994). Magnetic detection of a single action potential in *Chara corallina* internodal cells. Biophys. J..

[17] Corsini E, Acosta V, Baddour N, Higbie J, Lester B, Licht P, Patton B, Prouty M, Budker D (2011). Search for plant biomagnetism with a sensitive atomic magnetometer. J. Appl. Phys..

[18] Volkov AG (2006). Plant Electrophysiology: Theory and Methods.

[19] Volkov AG (2019). Signaling in electrical networks of the Venus flytrap (*Dionaea muscipula* Ellis). Bioelectrochemistry.

[20] Iosip AL, Böhm J, Scherzer S, Al-Rasheid KAS, Dreyer I, Schultz J, Becker D, Kreuzer I, Hedrich R (2020). The Venus flytrap trigger hair-specific potassium channel KDM1 can reestablish the K^+^ gradient required for hapto-electric signaling. PLOS Biol..

[21] Forterre Y, Skotheim JM, Dumais J, Mahadevan L (2005). How the Venus flytrap snaps. Nature.

[22] Sachse R, Westermeier A, Mylo M, Nadasdi J, Bischoff M, Speck T, Poppinga S (2020). Snapping mechanics of the Venus flytrap (*Dionaea muscipula*). PNAS.

[23] Suda H, Mano H, Toyota M, Fukushima K, Mimura T, Tsutsui I, Hedrich R, Tamada Y, Hasebe M (2020). Calcium dynamics during trap closure visualized in transgenic Venus flytrap. Nat. Plants.

[24] Scherzer S, Shabala L, Hedrich B, Fromm J, Bauer H, Munz E, Jakob P, Al-Rascheid KAS, Kreuzer I, Becker D, Eiblmeier M, Rennenberg H, Shabala S, Bennett M, Neher E, Hedrich R (2017). Insect haptoelectrical stimulation of Venus flytrap triggers exocytosis in gland cells. PNAS.

[25] Böhm J, Scherzer S, Krol E, Kreuzer I, von Meyer K, Lorey C, Mueller TD, Shabala L, Monte I, Solano R, Al-Rasheid KAS, Rennenberg H, Shabala S, Neher E, Hedrich R (2016). The Venus flytrap *Dionaea muscipula* counts prey-induced action potentials to induce sodium uptake. Curr. Biol..

[26] Palmer WM, Martin AP, Flynn JR, Reed SL, White RG, Furbank RT, Grof CPL (2015). PEA-CLARITY: 3D molecular imaging of whole plant organs. Sci. Rep..

[27] Böhm J, Scherzer S, Shabala S, Krol E, Neher E, Mueller TD, Hedrich R (2016). Venus flytrap HKT1-type channel provides for prey sodium uptake into carnivorous plant without conflicting with electrical excitability. Mol. Plant.

[28] Thiel F, Schnabel A, Knappe-Grüneberg S, Stollfuß D, Burghoff M (2007). Demagnetization of magnetically shielded rooms. Rev. Sci. Instrum..

[29] Budker D, Romalis M (2007). Optical magnetometry. Nat. Phys..

[30] Osborne, J., Orton, J., Alem, O. & Shah, V. Fully integrated, standalone zero field optically pumped magnetometer for biomagnetism. In *Proceedings on SPIE 10548, Steep Dispersion Engineering and Opto-Atomic Precision Metrology XI*, 105481G (2018).

[31] Scherzer S, Federle W, Al-Rasheid KAS, Hedrich R (2019). Venus flytrap trigger hairs are micronewton mechano-sensors that can detect small insect prey. Nat. Plants.

[32] Nguyen CT, Kurenda A, Stolz S, Chételat A, Farmer EE (2018). Identification of cell populations necessary for leaf-to-leaf electrical signaling in a wounded plant. PNAS.

[33] Volkov AG, Forde-Tuckett V, Reedus J, Mitchell CM, Volkova MI, Markin VS, Chua L (2014). Memristors in the Venus flytrap. Plant Signal Behav..

[34] Fagaly RL (2006). Superconducting quantum interference device instruments and applications. Rev. Sci. Instrum..

[35] Schnabel A, Burghoff M, Hartwig S, Petsche F, Steinhoff U, Drung D, Koch H (2004). A sensor configuration for a 304 SQUID vector magnetometer. Neurol. Clin. Neurophysiol..

[36] Liew LA, Knappe S, Moreland J, Robinson H, Hollberg L, Kitching J (2004). Microfabricated alkali atom vapor cells. Appl. Phys. Lett..

