# Supplementary material for: Action potentials induce biomagnetic fields in carnivorous Venus flytrap plants
Source: Sci Rep. 2021 Jan 14;11:1438. doi: 10.1038/s41598-021-81114-w (PMC7809347; doi:10.1038/s41598-021-81114-w)
Supplement: Supplementary file 2 — Supplementary Information. [file 41598_2021_81114_MOESM2_ESM.docx]

**Supplementary Information for**

**Action potentials induce biomagnetic fields in carnivorous Venus flytrap plants**

Anne Fabricant, Geoffrey Z. Iwata, Sönke Scherzer, Lykourgos Bougas, Katharina Rolfs, Anna Jodko-Władzińska, Jens Voigt, Rainer Hedrich, and Dmitry Budker

**Heat stimulation**

As part of our study of heat-induced flytrap electrical behavior, we compared the amplitude and depolarization kinetics of APs recorded at 10, 20, 30, and 40°C. There was a 1.6-fold increase in AP amplitude from 10 to 40°C. When heating the trap from 10 to 30°C, the half-depolarization time dropped from 0.29 ± 0.08 s to 0.13 ± 0.02 s.

**SQUID measurements**

In the PTB data run, as a complement to the OPM measurements we also conducted two types of experiments using 57 channels of the BMSR-2 built-in SQUID array. The first type involved placing an intact flytrap plant directly under the SQUID dewar, whose bottom surface has a 2.8-cm offset from the plane of the pick-up coils. We closed each trap in turn by two consecutive mechanical stimulations of the trigger hairs with a plastic pipette tip. In the data analysis, we looked for signals in the magnetic data corresponding to either the APs or subsequent trap closure. Even after averaging multiple SQUID channels, no signals were found, probably because of the large distance between sample and sensors. In the second type of SQUID experiment, we attached an isolated trap lobe directly to the bottom of the dewar and performed mechanical stimulation, but again no magnetic signals were found during data analysis. Following the calculation in the main body of the paper, at offset distance of at least 2.8 cm from the sample we would reasonably expect a magnetic-field magnitude on the order of 10 fT. Since this is approximately the noise floor of the SQUID magnetometer system at 1 Hz (under ideal operating conditions), the null result is consistent with expectations.

**Electrode tests**

Prior to the data run, we tested the electrode system to ensure that no spurious magnetic fields due to currents in the electrode wires would be picked up by the magnetometers under usual experimental conditions. These tests were conducted using the circuit depicted in Fig. S3, with a four-layer MS-2 magnetic shield from Twinleaf containing two active QuSpin sensors (B and C) placed side-by-side. A function generator (Tektronix AFG2021) in parallel with a resistor created a sawtooth “artificial flytrap action potential” signal at 1.2 Hz. This signal was sent through a low-noise voltage preamplifier (SRS Model SR560) with typical experimental settings (6-dB low-pass 10-Hz filter, AC coupling, gain 1000, input impedance 100 MΩ) that yielded a preamplifier output of amplitude 2 V—corresponding to the output amplitude we would see in an actual flytrap experiment. The crucial step was to simulate a “worst-case scenario” for the electrode wires. To that end, a 1-cm copper coil, in series with the preamplifier, was placed directly on top of sensor C. As is evident in Fig. S4, no signal at 1.2 Hz was visible in the y-axis or z-axis data of either magnetometer. Even when we increased the amplitude of the electric signal by five times (corresponding to 10-V preamplifier output), no signal at 1.2 Hz was observed. Thus, we were satisfied that the electrode/voltage-preamplifier system was not a source of unwanted noise. The voltage preamplifier and electronics used in these diagnostic experiments were the same as those used in BMSR-2 for plant experiments.

For comparison, we also conducted identical tests with a low-noise *current* preamplifier (SRS Model SR570, 6-dB low-pass 10-Hz filter, sensitivity 100 nA/V). In this case the signal at 1.2 Hz did appear above the noise in the data of sensor C. For example, the 2-V experiment yielded a 3-pT signal along the *y*-axis, indicating that a current of over 20 nA was flowing in the current loop. Based on these results, we exclusively used the voltage preamplifier in our data run at PTB. As an additional security check, in all OPM experiments we ran one of the electrode wires over the background sensor D to monitor for possible spurious signals (none were detected).

**OPM data**

Figure S5 shows the electric time traces used in the data analysis for Figs. 4 and 5. The recorded APs are slightly variable in shape and exhibit certain artifacts, which is normal for surface-electrode measurements. In some time traces (e.g. Fig. S5A) we observed the frequency of autonomous AP firing increasing over time, which may be explained as follows. Sufficient input energy is required to increase the cytosolic calcium level to threshold—once this threshold is reached, an AP is released. As the trap heats up in our setup, the stored cellular energy increases while the new energy which needs to be input for the next AP decreases, which could lead to an increase in AP firing frequency.

To characterize the performance of the QuSpin gradiometer system in the shielded room, we recorded the background in the room and performed frequency analysis. A typical noise spectrum is shown in Fig. S6.

**Corresponding author**

Correspondence to Anne Fabricant.

**SI Figures**


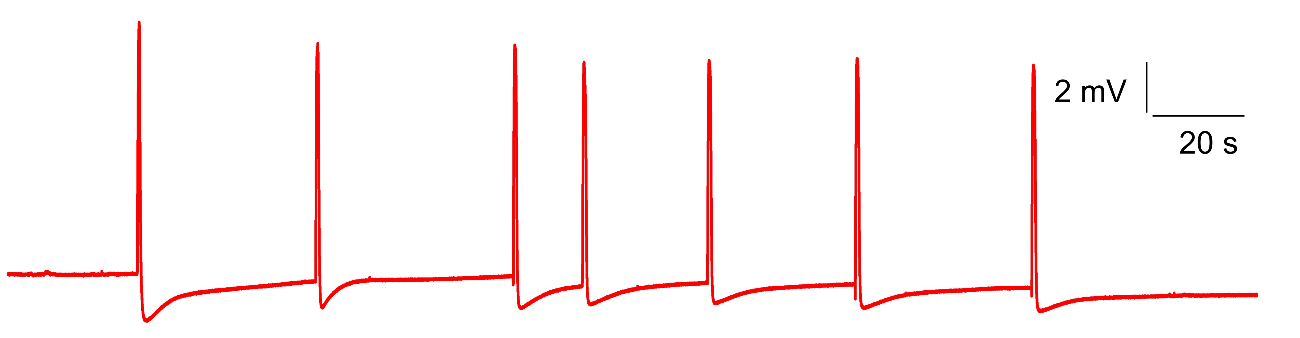


**Fig. S1.** Spontaneous AP firing on a hot plate heated to 46°C. Surface-potential measurements confirming that heat evokes APs. Corresponds to Movie S1, showing trap closure on the hot plate.


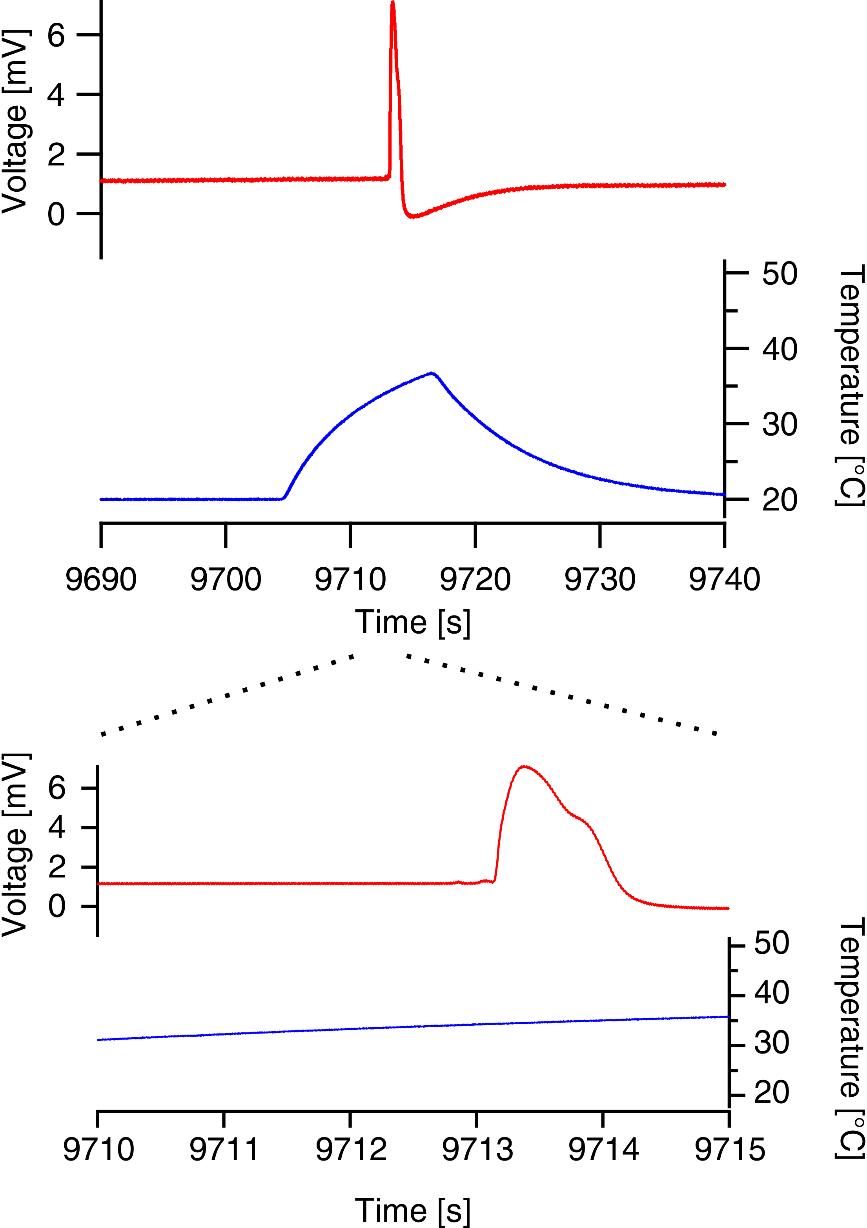


**Fig. S2.** Comparison of measured surface potential and applied temperature. Heat was applied via a Peltier element placed on the inner trap surface. An AP (red curve) occurred as the temperature (blue curve) increased from 20 to 45°C. The lower graph is a zoom-in on the time axis to define the temperature at which the AP occurred.


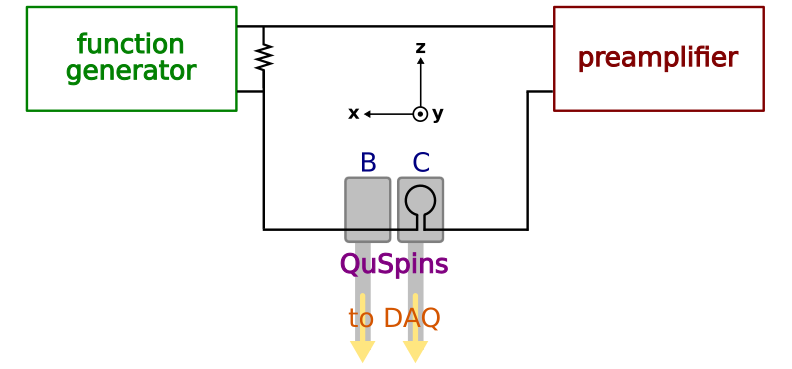


**Fig S3.** Circuit for testing the preamplifiers. See SI text for details.


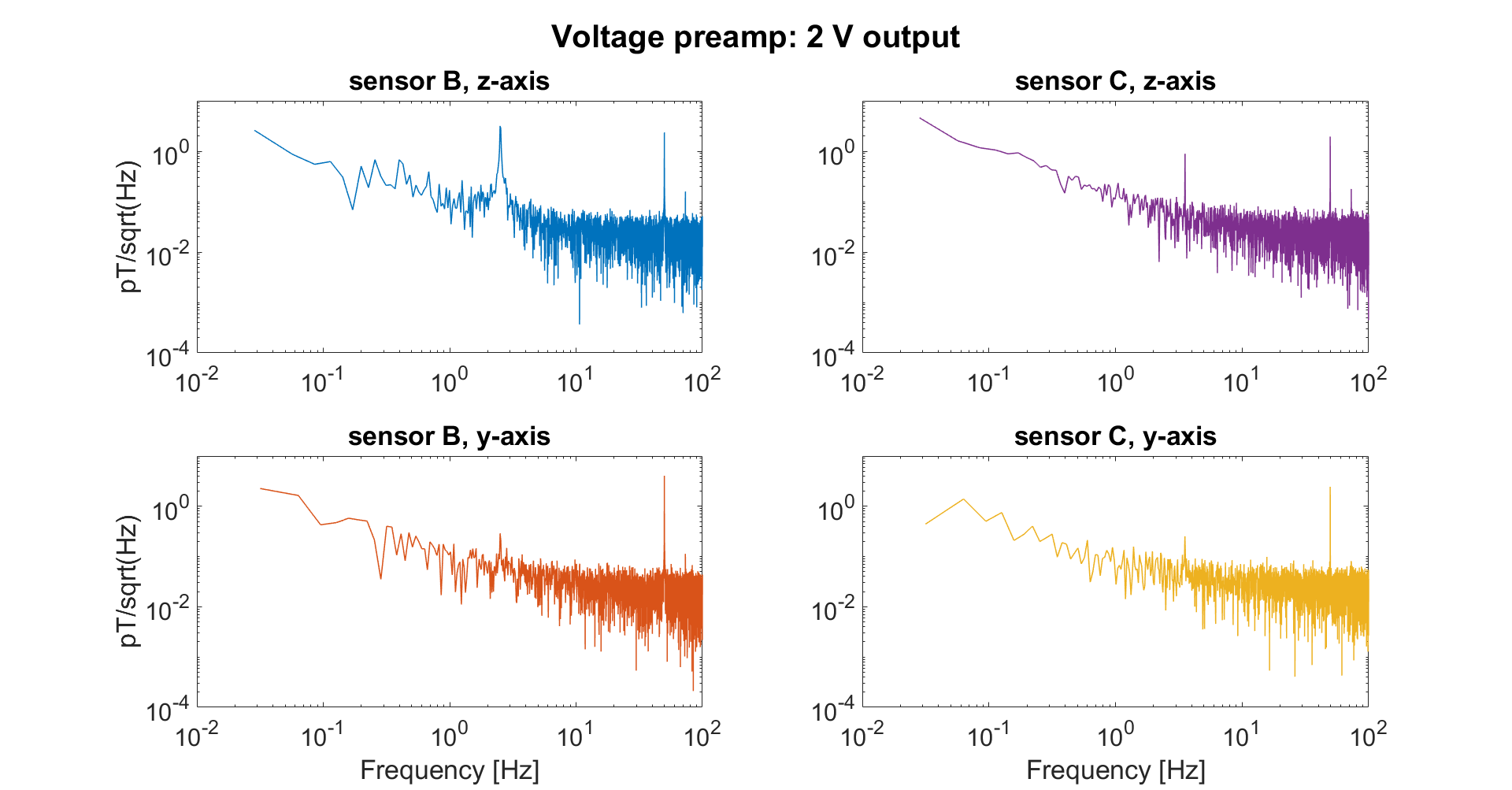


A


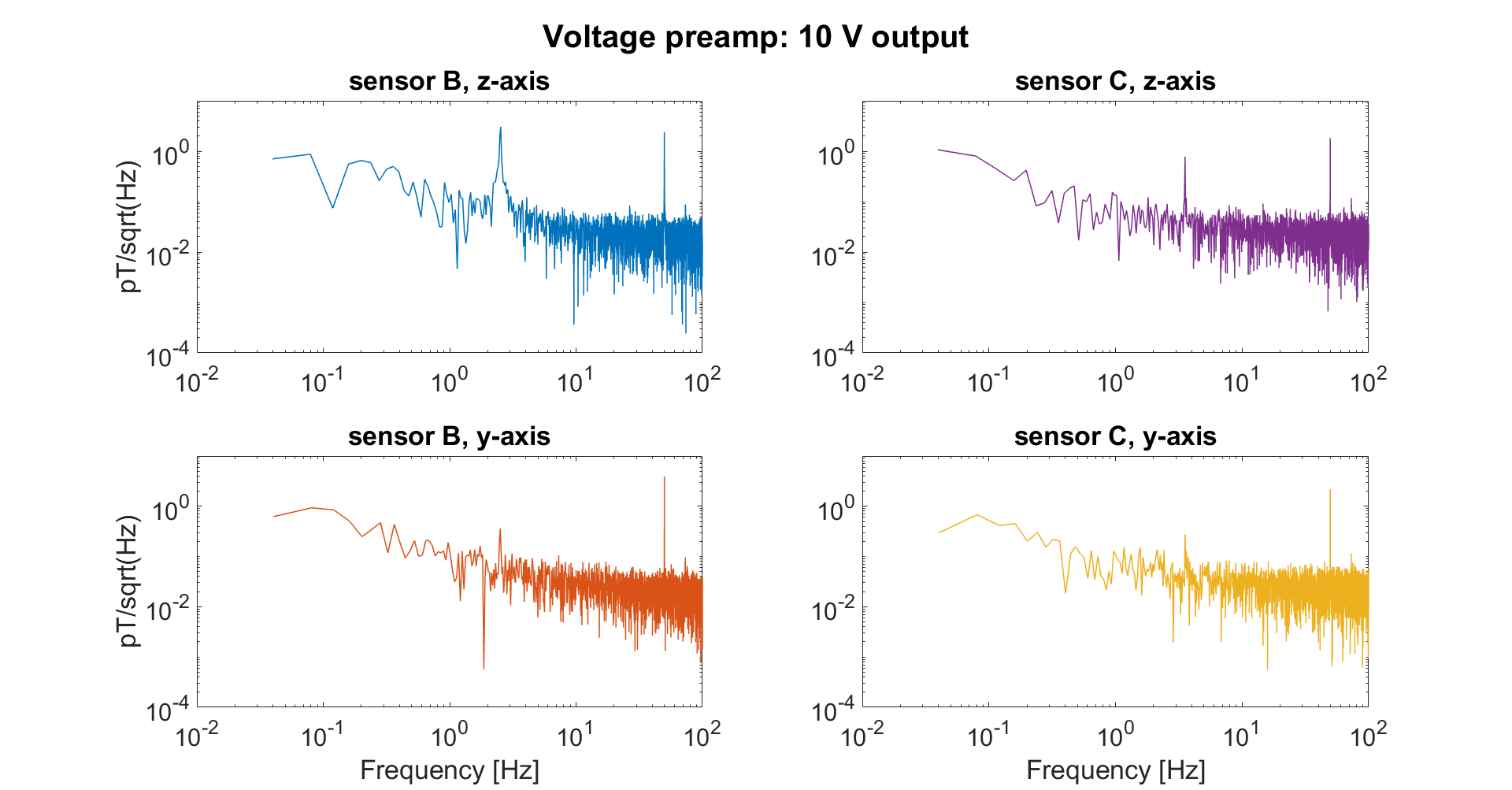


B


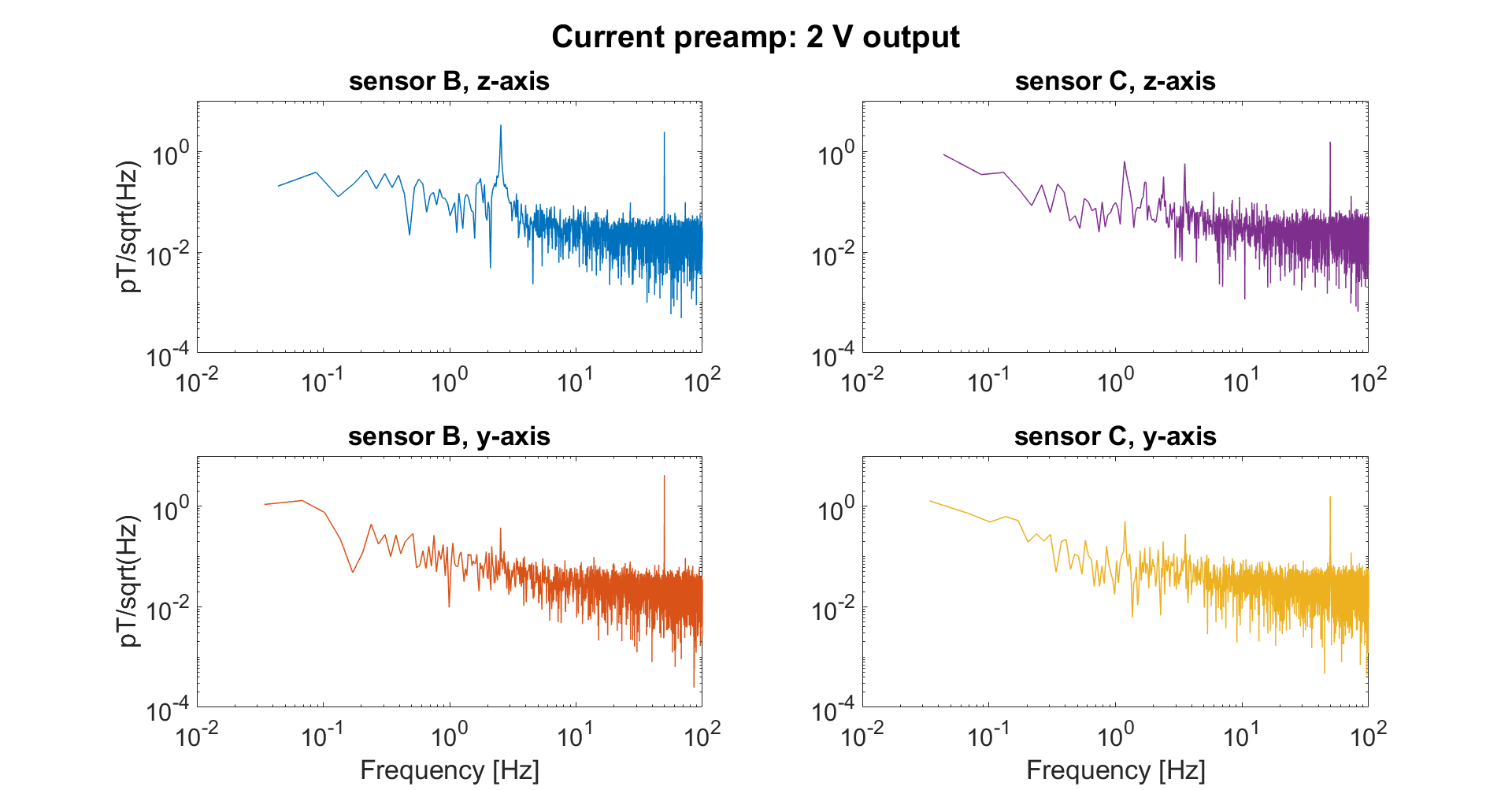


C


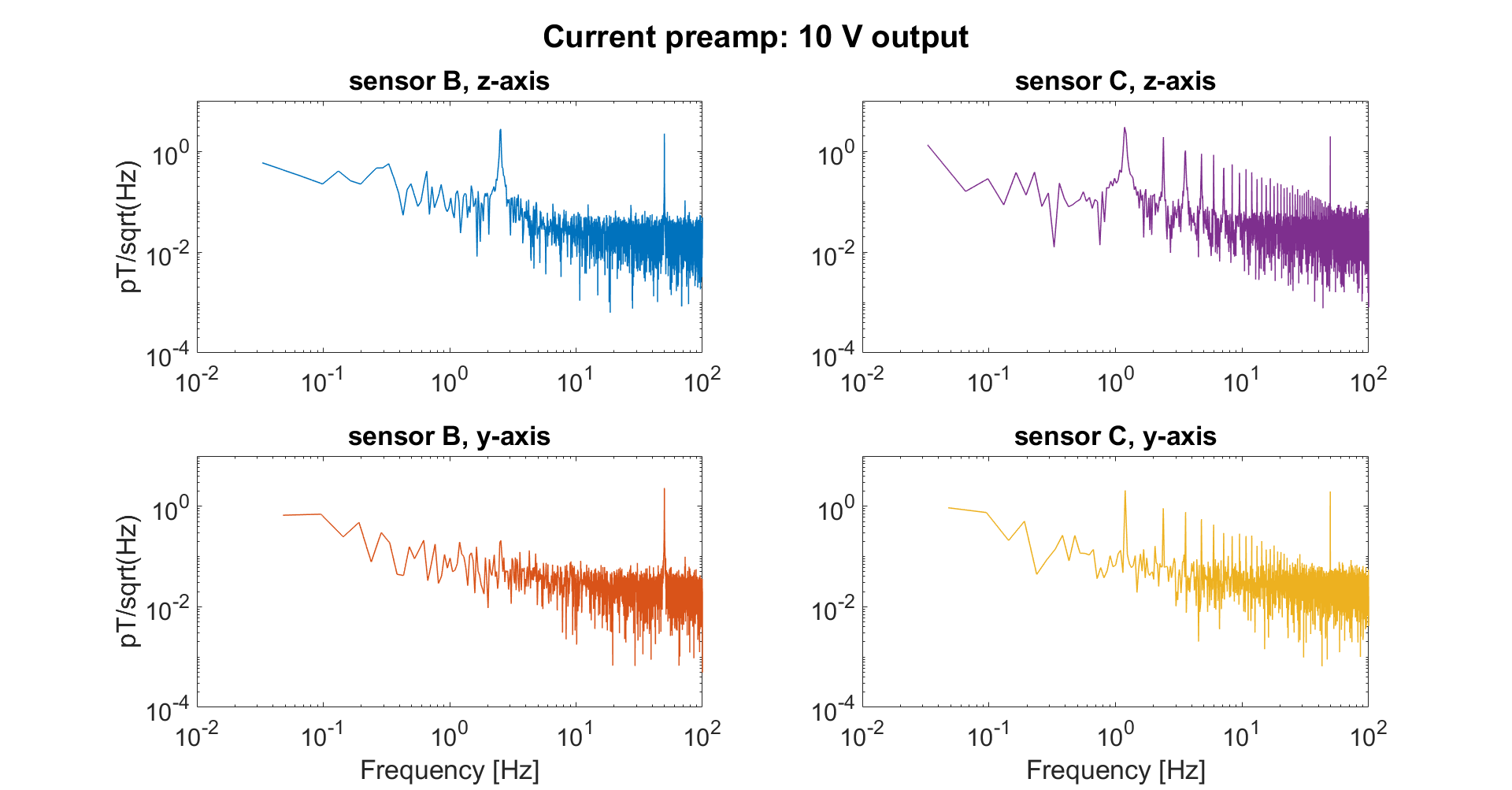


D

**Fig. S4.** Results of the preamplifier tests. In addition to the 50 Hz line frequency, peaks due to lab background noise appear at 2.5 and 3.5 Hz. Signal from the current preamplifier appears in the data of sensor C (C and D), but this effect is not seen when the voltage preamplifier is used (A and B).


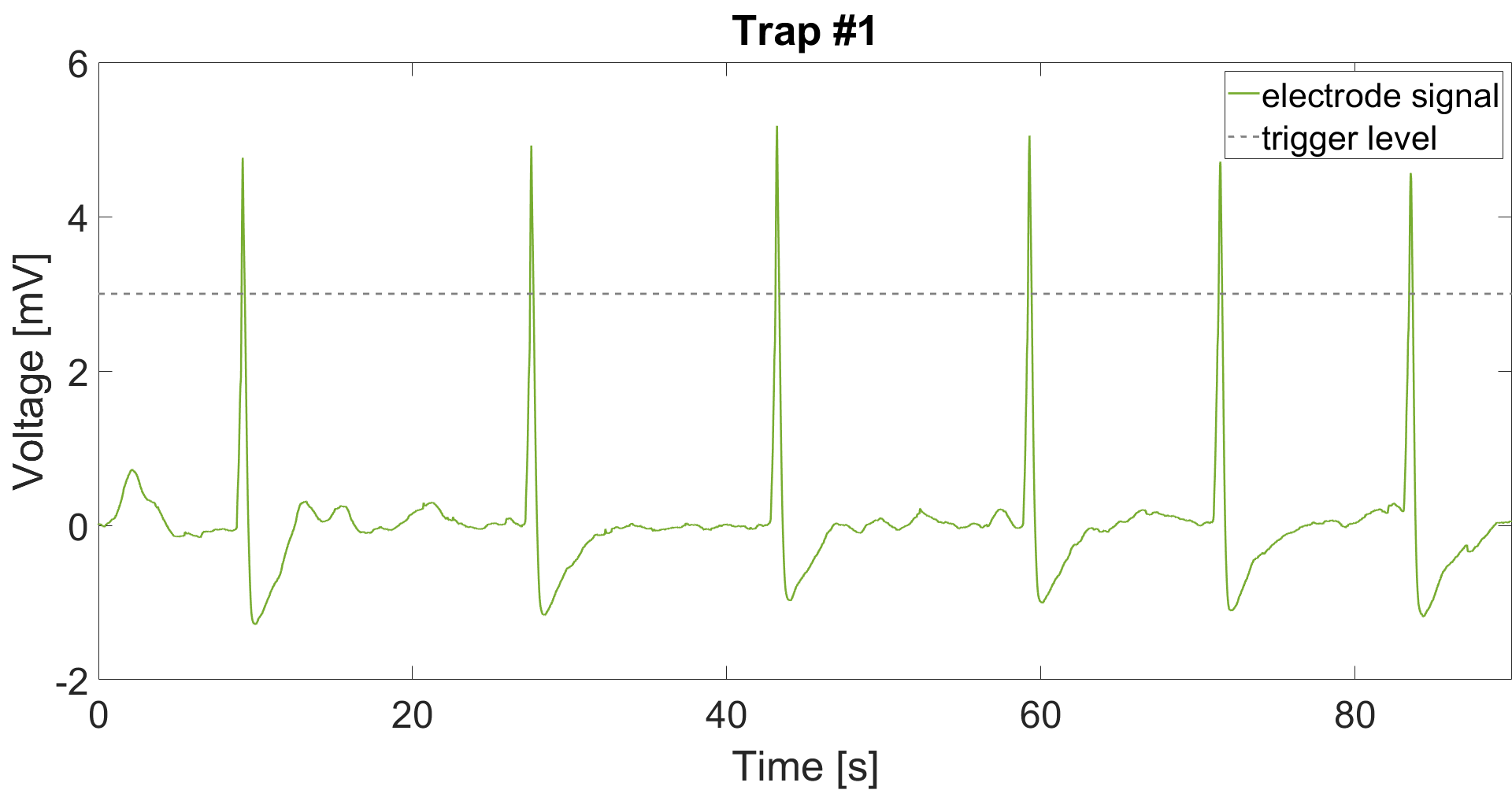


A

B


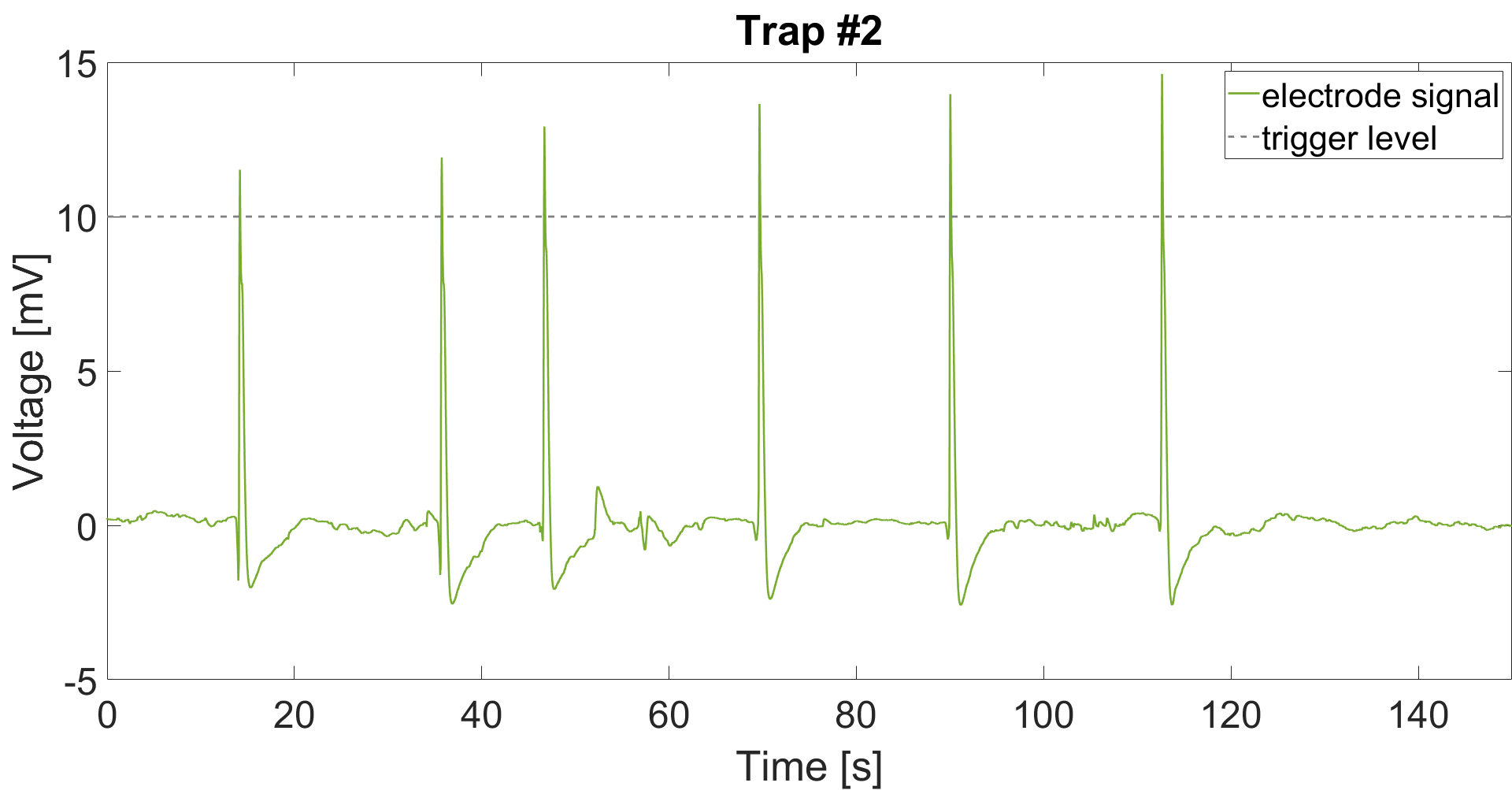


C


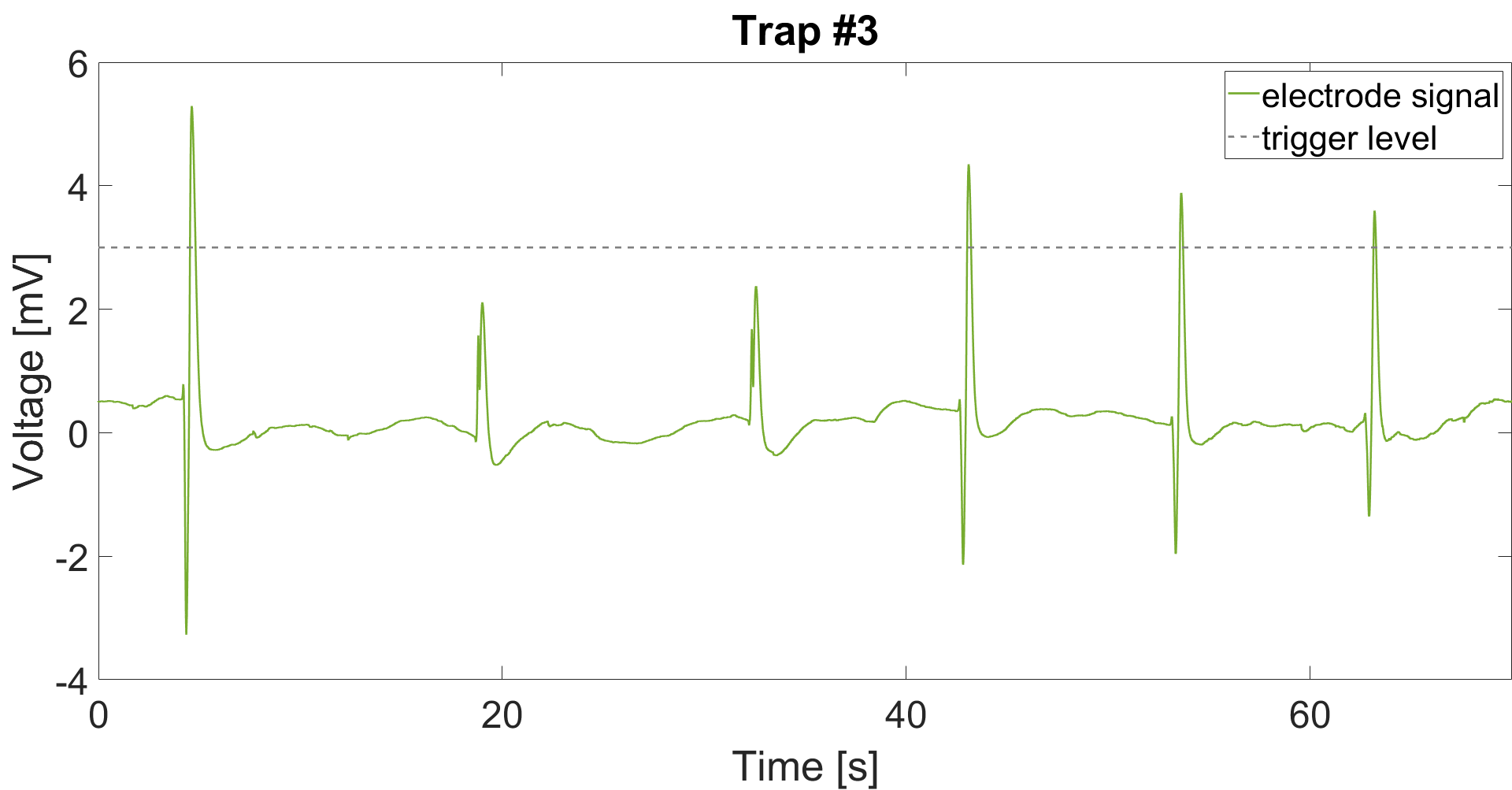


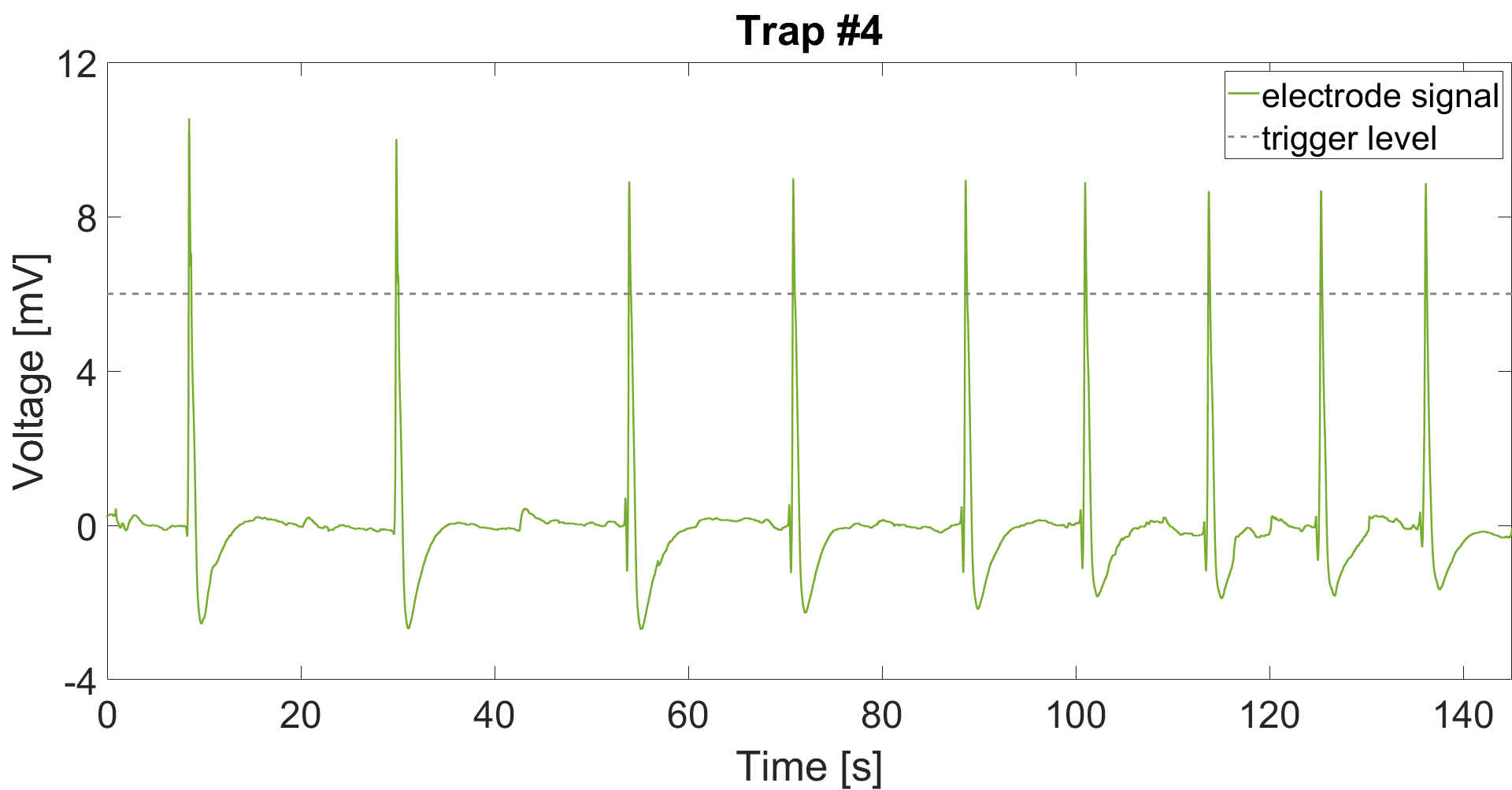


D

**Fig. S5.** Electric time traces showing heat-induced action potentials. Data were recorded from four separate experiments (A-D) with different plant samples, corresponding to the data shown in Fig. 5. The APs are used as a trigger so that we can perform averaging of the simultaneous magnetic data; the trigger level is indicated by the gray dashed line.


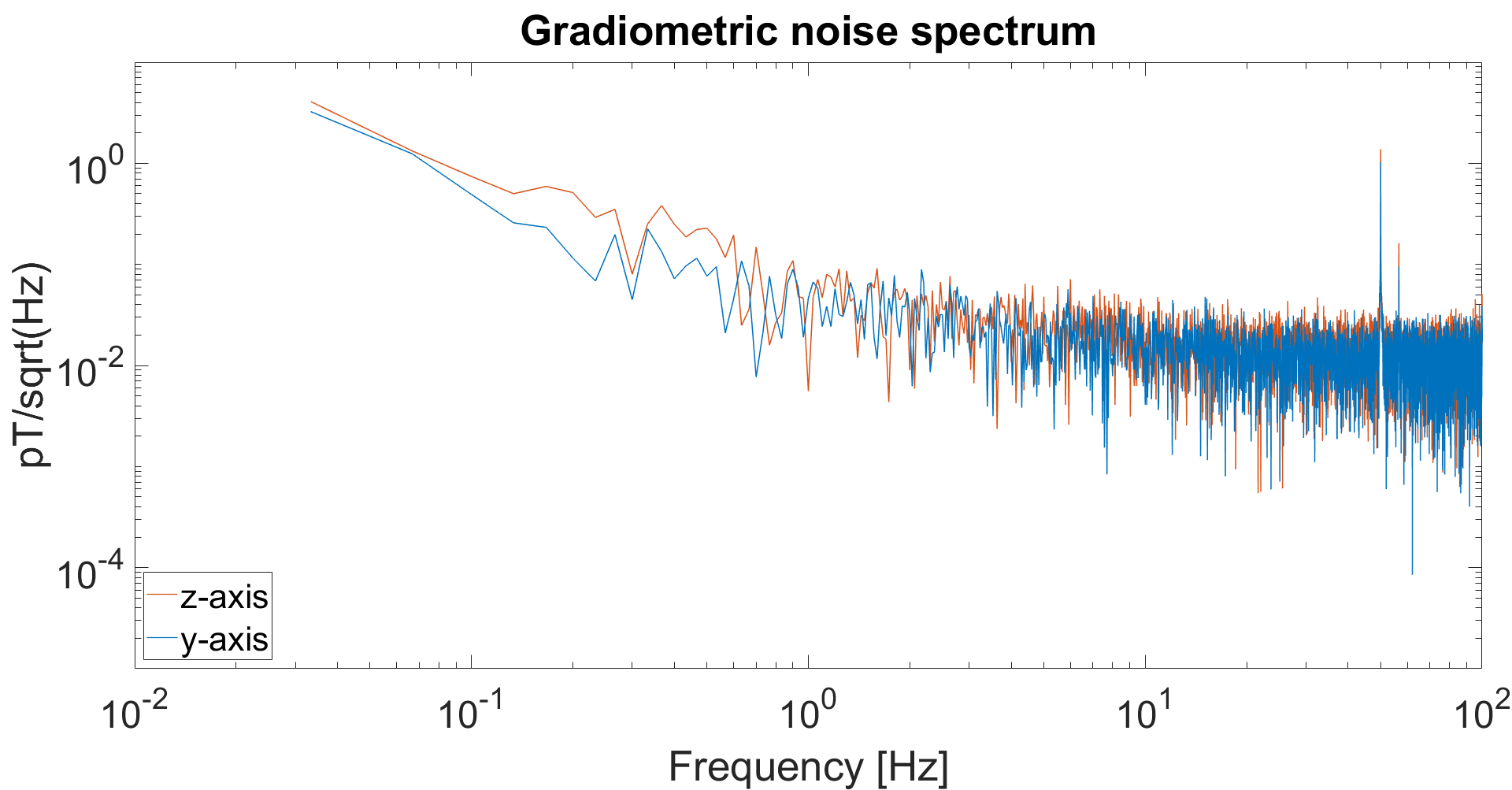


**Fig. S6.** Typical noise floor of the gradiometer in the magnetically shielded room. Obtained by recording a 30 s time trace prior to the start of an experiment.

**Caption for Movie S1 (separate file)**. Trap closure on a hot plate heated to 46°C. Playback speed is increased by a factor of 10. Corresponds to Fig. S1.
